# Pulmonary infections mimicking malignancy on bronchoscopy: A retrospective single‐center study in Japan

**DOI:** 10.1002/jgf2.383

**Published:** 2020-10-14

**Authors:** Naoya Itoh, Junichi Shimizu, Hiromi Murakami, Toyoaki Hida

**Affiliations:** ^1^ Division of Infectious Diseases Aichi Cancer Center Hospital Nagoya Japan; ^2^ Collaborative Chairs Emerging and Reemerging Infectious Diseases National Center for Global Health and Medicine Graduate School of Medicine Tohoku University Miyagi Japan; ^3^ Department of Thoracic Oncology Aichi Cancer Center Hospital Nagoya Japan

**Keywords:** bronchoscopy, cancer, infectious diseases, tuberculosis

## Abstract

**Background:**

Pulmonary infections can imitate pulmonary neoplasms. Pulmonary tuberculosis (TB) is a typical example of an infection that mimics cancer and results in unexpected exposure of healthcare workers to TB. A large number of patients with suspected lung malignancy are referred to cancer centers, although the epidemiology of the final diagnosis is unclear in Japan. This study aimed to determine the frequency and nature of pulmonary infections that imitate malignancy among patients with presumed lung cancer that is subsequently diagnosed as a pulmonary infection based on bronchoscopy findings. We also aimed to identify the prevalence of formerly undiagnosed pulmonary tuberculosis that could pose an occupational risk to healthcare workers.

**Methods:**

This single‐center retrospective cross‐sectional study included patients with suspected pulmonary malignancy who underwent bronchoscopy at a tertiary care cancer center in Japan between April 2017 and March 2020. Electronic medical records of the bronchoscopy database were reviewed to identify the final diagnoses recorded by physicians.

**Results:**

Among the 460 patients enrolled in the present study, 362 (78.7%) and 8 (1.7%) had primary or metastatic pulmonary lesions and benign lesions, respectively. Sixty‐six patients (14.3%) had nonspecific findings or other pulmonary diseases. Infection was confirmed in 24 patients (5.2%). Mycobacterial infections (n = 16) were the most frequent infectious disease; four patients had TB and 12 had nontuberculous mycobacterial infections.

**Conclusions:**

Despite the rare occurrence of TB in patients with suspected lung malignancy, healthcare workers should remain vigilant regarding the possibility of TB to prevent occupational exposure during invasive procedures such as routine bronchoscopy.

## Graphical abstract

There is no epidemiological information on the final diagnosis of patients suspected malignancy at Japanese cancer centers. Among patients referred for the confirmation of suspected pulmonary malignancy, 5.2% were confirmed to have lung infections, and 16.7% of these patients were unexpectedly found to have pulmonary TB. Our findings indicate the need to use a fit‐tested N95 particulate respirator to prevent occupational exposure to TB from patients with suspected malignancy during routine bronchoscopy.

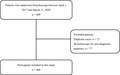

## 1 INTRODUCTION

Pulmonary infections can imitate pulmonary neoplasms. In particular, pulmonary tuberculosis (TB) is a leading example of an infection that mimics cancer^1^ and is well known as a diagnostic chameleon that can resemble a malignancy. TB and lung cancer present with similar clinical symptoms (fever, cough, sputum, hemoptysis, weight loss, anorexia, etc) and radiological findings.^2^

The rate of incidence of newly notified TB cases in Japan was 12.3 per 100 000 population in 2018,^3^ and therefore, Japan is categorized as an intermediate TB‐burden country similar to South Korea. Na et al reported that 76 of 1650 patients were unexpectedly diagnosed with TB after undergoing bronchoscopy for suspected nontuberculous pulmonary diseases between 2011 and 2013 at Pusan National University Hospital.^4^ Therefore, TB can result in the inadvertent exposure of healthcare workers to TB during diagnostic evaluation, especially during invasive diagnostic procedures such as bronchoscopy. The consensus statement of the American College of Chest Physicians and American Association for Bronchology included a recommendation that healthcare workers should wear a fit‐tested N95 particulate respirator to minimize exposure to airborne TB during bronchoscopy of patients with suspected pulmonary TB.^5^ There are several reports from other countries with regard to lung infections that mimic cancer, although some of them are endemic fungal infections and the epidemiology differs from that in Japan.^6^, ^7^ Rolston et al reported pulmonary infections that mimic cancer at a US cancer center, wherein 46% of the infections were endemic fungal infections (eg, histoplasmosis, cryptococcosis, and coccidioidomycosis).^6^ Similarly, Homrich et al reported lung infections that mimic cancer in a Brazilian university hospital, and approximately 20% of the infections were endemic mycoses.^7^ In Japan, reports of these abovementioned infections are incredibly scarce.^8^

Thus, this study was conducted to determine the frequency and nature of pulmonary infections that imitate malignancy among patients with presumed lung cancer that is subsequently diagnosed as a pulmonary infection based on findings from bronchoscopy. A secondary objective of our study was to identify the prevalence of formerly undiagnosed pulmonary tuberculosis that could pose an occupational risk to healthcare workers.

## 2 PATIENTS AND METHODS

This retrospective analysis covering a 3 year period enrolled patients who were referred to our Cancer Center Hospital, Japan—a 500‐bed tertiary care hospital wherein approximately 11 000 patients are admitted per year. We extracted information from the bronchoscopy database for all adult patients (age ≥ 18 years) who underwent bronchoscopy between April 1, 2017, and March 31, 2020. The electronic medical records of the bronchoscopy database were reviewed, and the final diagnoses recorded by the physicians were identified and collated. Patients who underwent bronchoscopy for nondiagnostic purposes (treatment and observation only) and duplicate patients (patients who underwent more than two bronchoscopies) were excluded. This study was approved by the institutional ethics committee, and all patient information was anonymized before the analysis. The need for consent was waived by the approving authority.

### 2.1 Data collection

The following patient information was extracted from the medical records: age, gender, histologic diagnosis, symptoms of pulmonary infection, causative organisms, and indications for bronchoscopy.

### 2.2 Statistical analysis

All data analyses were conducted using SPSS version 18 (SPSS Inc).

## 3 RESULTS

**FIGURE 1:**
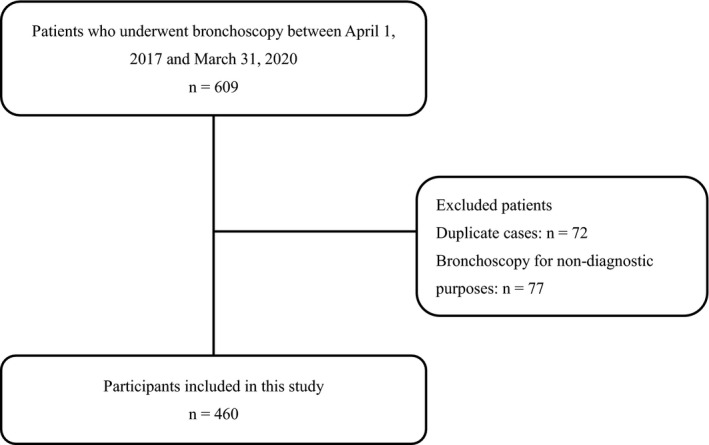
Flow diagram of patient selection in this study

We screened 609 patients for inclusion and excluded 149 based on the prespecified study eligibility criteria (Figure 1). The final analysis dataset included 460 patients (median age 70 years, interquartile range 63‐76 years; males 63.3%). Among these 460 patients, 421 (91.5%) were outpatients and 39 (8.5%) were inpatients. Table 1 presents the distribution of the final diagnoses. Of the patients who underwent diagnostic evaluation, 362 (78.7%) had either a primary or metastatic pulmonary lesion, 8 (1.7%) had a benign lesion, and 24 (5.2%) had an infection. Infections in patients were diagnosed using sputum samples collected by endotracheal suctioning in 21 patients and brush cytology in 3 patients. Among patients with infections, two concurrently had nontuberculous mycobacterial (NTM) infections and malignancy. Sixty‐six patients (14.3%) had nonspecific findings or other pulmonary diseases.

**Table 1:** Final diagnosis of 460 patients who underwent bronchoscopy

Final diagnosis	No. (%) of patients
Malignancy (primary or metastasis)	362 (78.7)
Benign lesion^a^	8 (1.7)
Infection	24 (5.2)
NTM infection	8
NTM infection plus malignancy	2
NTM infection plus aspergillosis	1
NTM infection plus bacterial pneumonia	1
Tuberculosis	4
Lung abscess or pneumonia	6
Aspergillosis	1
Other pulmonary disease/nonspecific findings	66 (14.3)

Abbreviations: NTM, nontuberculous mycobacteria.

^a^ Eight patients with benign lesions were included: sarcoidosis (n = 3), Castleman's disease (n = 2), and others (n = 2).

**FIGURE 2:**
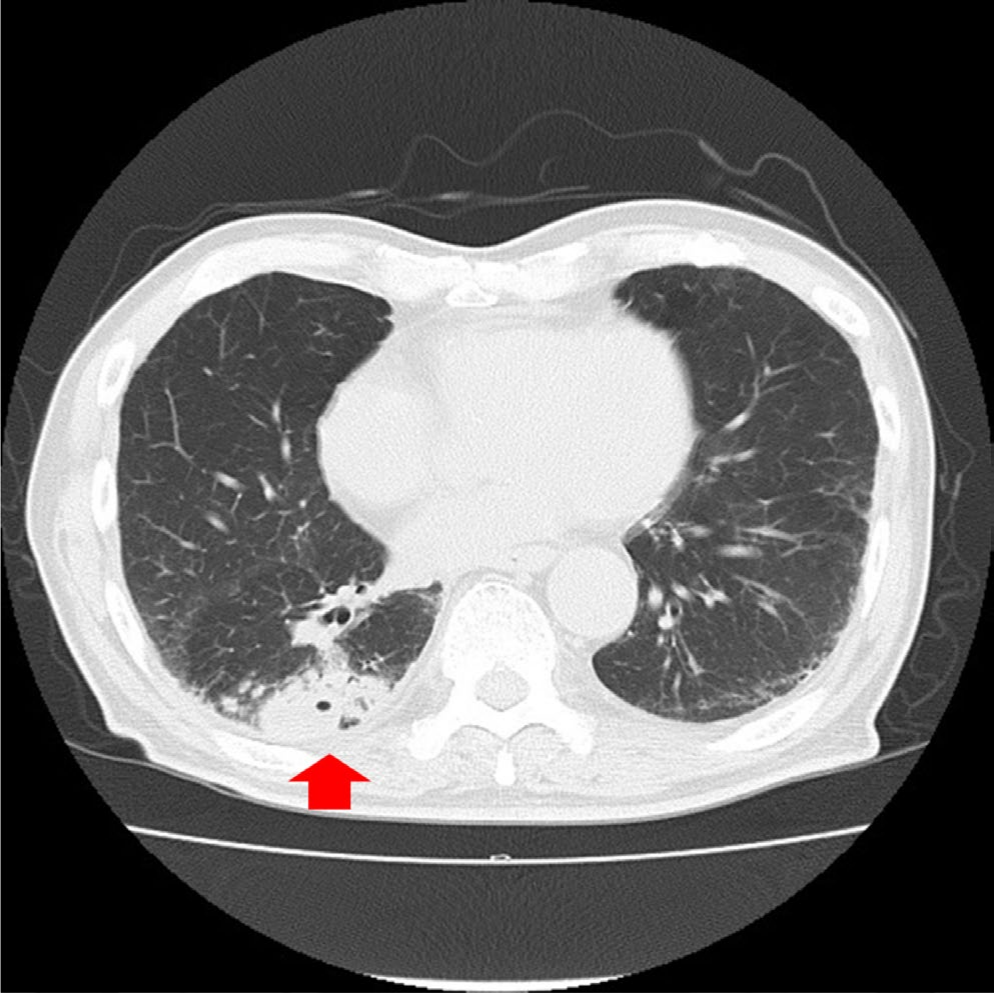
Chest computed tomography (CT) scan of an 85‐y‐old male with intraductal papillary mucinous neoplasms (IPMN) of the pancreas showing a mass with a cavity and surrounding nodules in the right lower lobe, which was diagnosed as *Mycobacterium tuberculosis*

**FIGURE 3:**
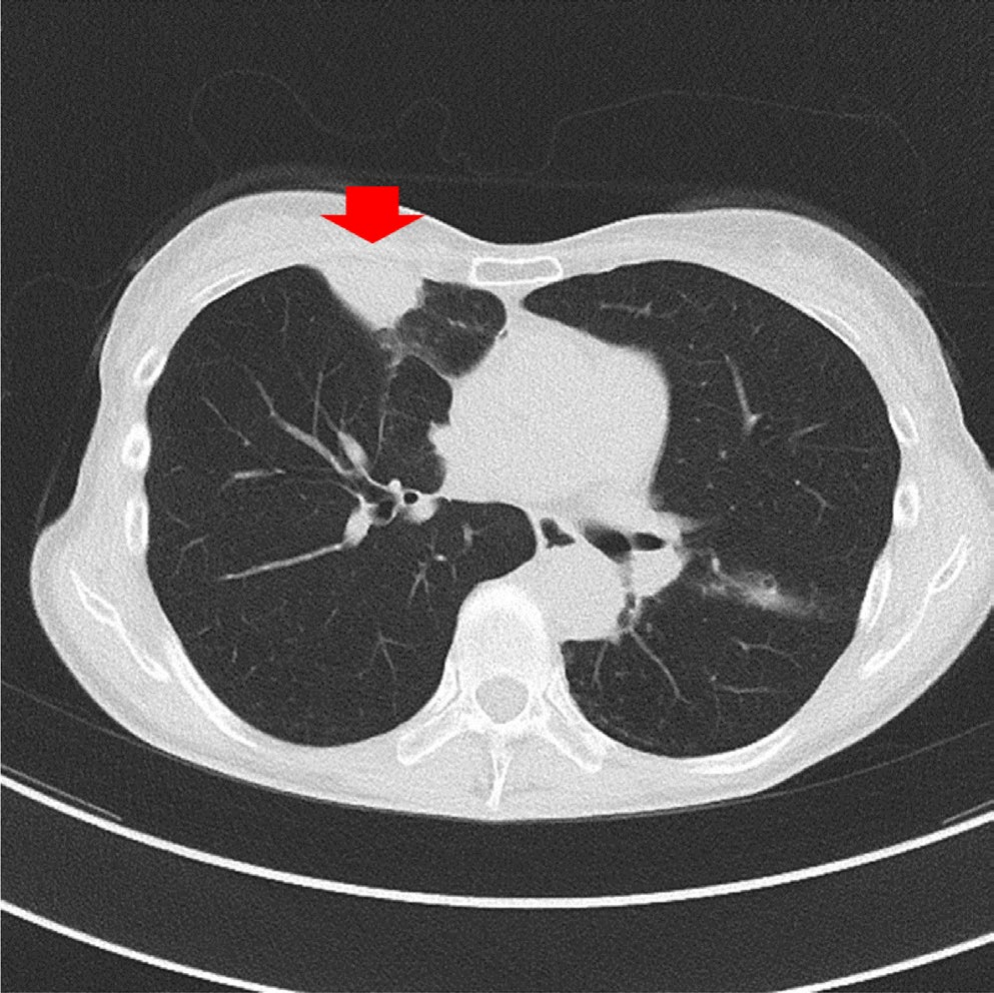
Chest computed tomography (CT) scan of a 68‐y‐old female with postoperative left upper lobe lung cancer. A large lesion is seen in the right middle lobe, which was diagnosed as *Mycobacterium avium* using sputum that was collected by endotracheal suctioning

**FIGURE 4:**
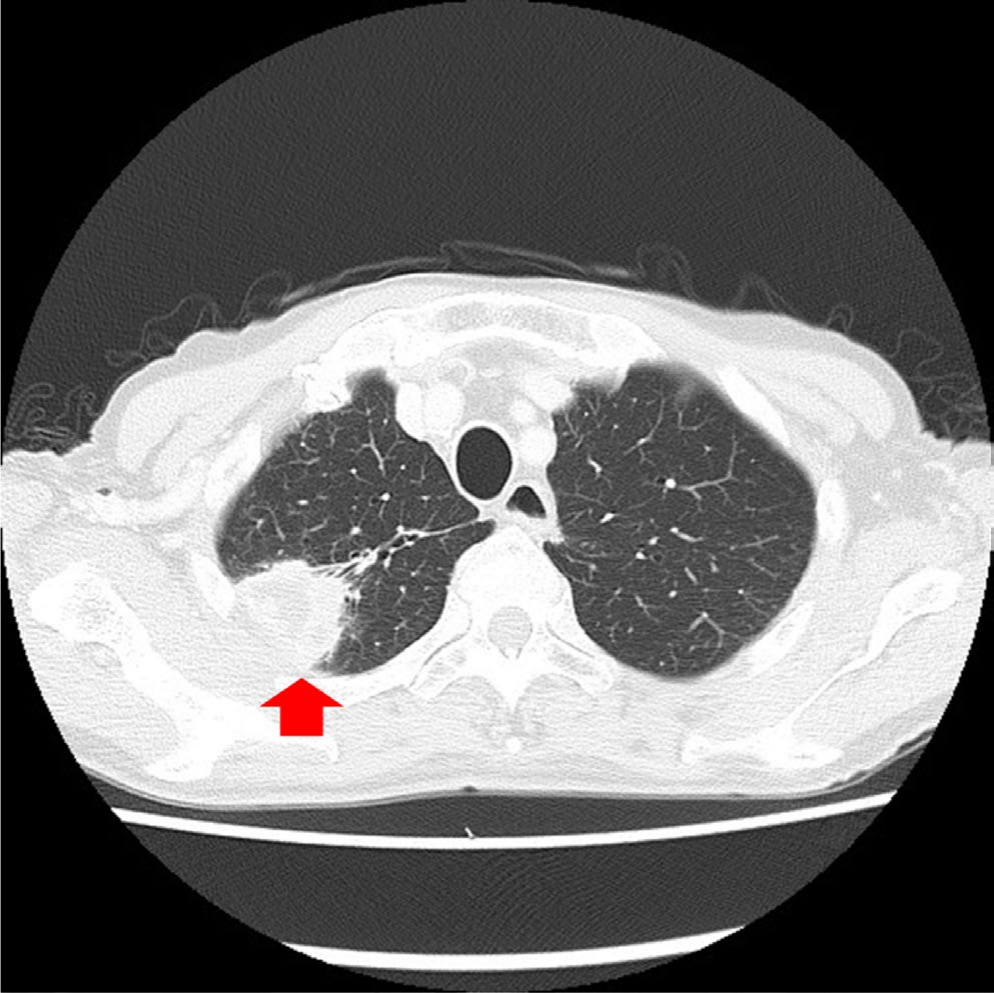
Chest computed tomography (CT) scan of an 81‐y‐old male showing a mass on the right pulmonary apex, which was diagnosed as a lung abscess caused by *α‐Hemolytic streptococci*, *Neisseria* spp., *Haemophilus* spp., and *Serratia fonticola*

Mycobacterial infections were the commonest infective disease and accounted for 16 (66.7%) of the 24 patients with infections and no malignancy. Among patients with mycobacterial infections, 4 (16.7%) had tuberculosis and 12 (50.0%) had NTM infections (Figures 2 and 3), which included aspergillosis (n = 1) and bacterial pneumonia (n = 1). Six patients had lung abscess or pneumonia (Figure 4). Table 2 presents the microbiological details at initial presentation for these 24 patients. Among these 24 patients, 12 (50.0%) were asymptomatic and the remaining 12 presented with various respiratory symptoms, with cough being the most common symptom (33.3%).

**Table 2:** Microbiological details of 24 patients with pulmonary infections

Type of infection	N (%) of isolated organisms
Mycobacterial	16 (41.0)
*Mycobacterium tuberculosis*	4
*Mycobacterium avium*	8
*Mycobacterium intracellulare*	2
*Mycobacterium* spp.	2
Fungal	2 (5.1)
*Aspergillus fumigatus*	1
*Aspergillus* spp.	1
Bacterial (excluding mycobacteria)	21 (53.8)
*α‐Hemolytic streptococci*	6
*Neisseria* spp.	2
*Corynebacterium* spp.	2
*Haemophilus* spp.	2
*Enterobacter cloacae*	2
*Streptococcus pneumoniae*	1
*Escherichia coli*	1
*Streptococcus haemolyticus*	1
*Cronobacter sakazakii*	1
Methicillin‐resistant *Staphylococcus aureus*	1
γ‐hemolytic *Streptococcus*	1
*Serratia fonticola*	1

Note

Eight patients had infections that were caused by multiple organisms.

## 4 DISCUSSION

In cancer centers in Japan, such as this study center, many patients are referred for the evaluation of a presumptive diagnosis of lung cancer (most often, based on routine radiographic studies). However, it is important to clarify the frequency and nature of other diseases that imitate cancer. We found that among patients referred for the confirmation of suspected pulmonary malignancy, 24 (5.2%) patients were confirmed to have lung infections. Of these 24 patients, 4 (16.7%) were unexpectedly found to have pulmonary TB.

The frequency of lung infections that mimic cancer was low, at 5.2%, in this study. The spectrum of these infections varies geographically and may be dependent on the regional prevalence of a particular infection. These findings suggested that pulmonary mycobacterial infections can mimic lung malignancies. Not only does pulmonary TB mimic lung malignancies ^1^, ^2^, but also NTM infections can imitate lung malignancy.^12^ Hong et al reported that NTM pulmonary infections may manifest as a solitary nodule, mass, or mass‐like consolidation that resembles a malignancy.^12^

In this study, mycobacterial infections, including TB and NTM, were the most prevalent infectious disease diagnosed, in 4 (16.7%) and 12 (50.0%) patients, respectively. A recent study from Tanzania reported a similar prevalence of TB in patients undergoing bronchoscopy^9^. As Japan is known to be an intermediate TB‐burden^3^ and a high NTM‐burden country (incidence of 12.3 per 100,000 population in 2018),^10^, ^11^ our findings indicate the need to use a fit‐tested N95 particulate respirator or higher‐grade respiratory precautions to prevent occupational exposure to TB from patients with suspected malignancy during routine bronchoscopy.

In this study, patients with infection were mostly asymptomatic or had a cough, which made it difficult to differentiate the infections from malignancy.

In this study, two patients had NTM infections that coexisted with malignancy. Taira et al reported that NTM infections were detected in 2.0%–8.5% of patients with lung cancer, and lung cancer and NTM infection rarely coexisted.^13^ Therefore, when performing bronchoscopy, it is important to undertake bronchial brushing and lavage and a transbronchial lung biopsy (TBLB) to differentiate infection from other diseases. However, positive cultures from bronchial lavage fluids do not exclude the possibility of complicated malignancies.

Other studies have found endemic fungal infections, such as coccidioidomycosis and histoplasmosis, in pulmonary lesions;^6^, ^7^ however, there were no endemic mycoses detected in this study. This difference might be attributable to the epidemiological differences in fungal infections,^8^ although the incidence of imported mycoses has recently been increasing in Japan.^14^

There were 66 patients (14.3%) with other pulmonary disease or nonspecific findings in this study. This result does not imply that lung cancer or infection was ruled out in all patients because of the possibility of biopsy errors. Chechani et al reported that TBLB had a diagnostic sensitivity of 57% for lung cancer, which suggested the likelihood of biopsy error.^15^ Thus, in the results of this study, we may have underestimated the incidence of malignancies and other infections.

Furthermore, the results showed that six patients with lung abscess and bacterial pneumonia had clinical findings that mimicked those of a malignancy. Moreover, this study showed that most of the infective bacteria were oral commensals. The main causative organisms of pneumonia and lung abscess that were reported from previous studies differ from those identified in this study.^16^, ^17^ However, it is unclear whether the bacteria that were identified were the actual causative organisms, as some of the patients in this study were treated with antimicrobials before bronchoscopy. A further possibility is that the bacteria identified may be as a result of sample contamination and not actual infections.

This study had some limitations. First, as the study center is an oncology hospital, the prevalence of infectious diseases is likely to be underestimated. Second, there is a possibility that an appropriate culture specimen may not have been provided and infections may have been missed. Third, there exists the possibility that an appropriate specimen may not have been collected on bronchoscopy. Finally, this was a single‐center study and, therefore, may not represent the nationwide characteristics from Japan. Thus, multicenter studies are warranted to obtain more information on the epidemiology of pulmonary infections presenting as malignancies in Japan. However, this study's strength is that this is the first research to describe the epidemiology of infections that mimic cancer at Japanese cancer centers.

In conclusion, tuberculosis was diagnosed in 4 (0.9%) of the 460 patients who underwent bronchoscopy during a 3 year study period. Healthcare workers should consider the use of higher‐grade respiratory precautions, such as a fit‐tested N95 particulate respirator, to prevent occupational exposure to TB during routine bronchoscopy.

## CONFLICT OF INTEREST

The authors have stated explicitly that there are no conflicts of interest in connection with this article.

## AUTHOR'S CONTRIBUTIONS

NI contributed to the study conception and design; NI and YM prepared the materials and undertook data collection and analysis; NI wrote the first draft of the manuscript; and JS, YM, and TH interpreted the data and assisted in the review of the final manuscript. All authors critically reviewed the draft of the manuscript and read and approved the final manuscript.

## ETHICAL APPROVAL

All procedures performed in this study were in accordance with the ethical standards of the Aichi Cancer Center Hospital Ethics Review Board and with the principles evinced in the 1964 Declaration of Helsinki and its later amendments or comparable ethical standards.

## AUTHORSHIP STATEMENT

All authors meet the ICMJE authorship criteria.

## Supporting information

App S1

## Data Availability

The dataset used and/or analyzed in this study are available in the Supporting Information.
